# RSV pre-fusion F protein enhances the G protein antibody and anti-infectious responses

**DOI:** 10.1038/s41541-022-00591-w

**Published:** 2022-12-19

**Authors:** Caixia Su, Yiwei Zhong, Gan Zhao, Jiawang Hou, Shuren Zhang, Bin Wang

**Affiliations:** 1 Key Laboratory of Medical Molecular Virology (MOE/NHC/CAMS), School of Basic Medical Sciences, Fudan University, Shanghai, China; 2 Fudan-Advaccine Join-Lab for Vaccine Research, Fudan University, Shanghai, China; 3 Shanghai Institute of Infectious Disease and Biosecurity, Shanghai, China; 4 Advaccine Biopharmaceutics (Suzhou) Co. LTD, Suzhou, Jiangsu Province China; 5 National Clinical Research Center for Geriatric Diseases, Huashan Hospital, Shanghai, China; 6 Children’s Hospital of Fudan University, Shanghai, China; 7 Present Address: Shenzhen Pregene Biopharma Company LTD, Shenzhen, China

**Keywords:** Protein vaccines, Viral infection

## Abstract

Respiratory syncytial virus (RSV) infection in children is the most common viral respiratory infection and can cause severe lung damage or death. There is no licensed vaccine for preventing RSV infection. Previously we demonstrated that an RSV vaccine, BARS13, consisting of recombinant G protein from *E. coli* plus cyclosporine A (CsA) as an immune-modulator, can protect animals from RSV challenge without inducing vaccine-enhanced disease (VED). To maximize the efficacy of such a vaccine, we introduced RSV pre-fusion F protein (pre-F) to form a new vaccine comprised of the pre-F and G proteins with the CsA. Two intramuscular immunizations with the vaccine induced a higher level of neutralizing antibodies against RSV and protected mice from RSV challenge without incurring VED. Interestingly, the addition of the pre-F to the vaccine facilitated anti-G antibody production and protection from RSV infection mainly via induction of antibodies against the central conserved domain (CCD) of the G protein which correlated with blocking the CX3C-CX3CR1 interaction. A 15 amino acid sequence (FP4) within the F2 region of pre-F served as a CD4^+^ Th epitope to facilitate the anti-G antibody response. Collectively, such a combination of the FP4 peptide with the G protein and CsA provides a novel strategy for developing a safe and maximally effective recombinant G protein-containing RSV vaccine.

## Introduction

RSV is the leading cause of acute lower respiratory tract infection in infants and young children. Although almost every infant under 2 years old experiences an RSV infection^1^, reinfections occur commonly throughout life and induce the most severe morbidity in children aged <5 years, older adults, and subjects with underlying conditions. Palivizumab has been used for prophylaxis of high-risk infants, but there is no licensed vaccine for treatment or protection against RSV^2,3^. A formalin-inactivated virus, FI-RSV, the earliest vaccine for RSV, was tested in the 1960s in clinical trials, but it resulted in vaccine-enhanced disease (VED) because of Treg loss and excessive Th2-dependent inflammation^4–6^.

The G glycoprotein of RSV is responsible for attaching the virus to the host cell’s surface to initiate the infection process. Importantly, non-glycosylated G protein produced by *E. coli* can induce an effective immune response with the assistance of adjuvants and VED is not triggered upon subsequent RSV challenge^7,8^. G protein contains a central conserved domain (CCD) located at the amino acid position aa159–191 that is shared between the serum types A and B. A previous study found that the CCD region is the immunodominant region since mice immunized with peptide sequences from other regions of the G protein did not induce antibodies against RSV G^9,10^. A variety of monoclonal antibodies (MAbs) against RSV G were discovered with specificity for aa164–176, including 131-2G and 3D3, that had neutralization ability against the RSV infection^11–13^. These mAbs could prevent RSV infection in cultured cells and murine models^13–18^. Thus, the sequence has been termed a neutralizing epitope on RSV G protein. The CX3C motif (aa182–186) within CCD was found to be crucial for RSV infection, and mutation of this motif significantly reduced viral infection^14,19–22^. This led to the discovery of CX3CR1, a surface receptor on the host cell that mediates RSV infection^23,24^. In addition, the CX3C motif could induce migration of CX3CR1^+^ lymphocytes to the infection site via a chemo-trafficking mechanism in the airway and lung, which resulted in increased severity of inflammation during the RSV infection^14,24–26^. Previous reports have demonstrated that the 131-2G mAb and antisera from mice immunized with the G protein could significantly inhibit the G protein binding to the CX3CR1 at position 293.CX3CR1, and further reduce the severity of RSV infection^9,10,27,28^. Thus, the G protein became an important target for RSV vaccine development. A previous report from our laboratory showed that immunization with the *E. coli*-produced recombinant G protein combined with CsA (named BARS13) induced a protective response against the RSV infection without VED by inducing anti-G neutralizing antibodies and Treg cells^8^. The BARS13 vaccine has completed a phase 1 clinical trial (manuscript in preparation) and is being tested in a phase 2 trial currently.

The RSV F glycoprotein is a fusion protein that mediates virus penetration and fusion between neighboring cells to form prominent syncytia through its six-fold coiled helix. The F protein is the most important target for neutralizing antibodies. The F protein is transformed from a metastable pre-fusion conformation (pre-F) to a stable post-fusion structure (post-F) during the fusion process^29,30^. The transition in conformation changes the neutralization epitopes on the fusion protein. Some epitopes are conserved in both pre-F and post-F, whereas others are only present in pre-F, for example, site Φ and V^23,31^. Numerous studies have revealed that the pre-F can induce higher neutralizing antibodies than the post-F and is expected to provide more effective protection against RSV infection^29,30,32–34^. Van der Fits L. et al. found that immunization with an Ad26 vector encoding the RSV pre-F protein could induce a protective response and long-lasting Th1-biased immunity in neonatal mice^35^.

The two most critical factors in developing a highly effective RSV vaccine are the elicitation of a high level of neutralizing antibodies and the avoidance of VED induction. We previously showed that vaccination with the BARS13 vaccine led to minimal VED production in a murine model. Since pre-F induced higher neutralizing antibodies in general, we sought to examine if stronger neutralizing antibodies against RSV infection could be elicited, while still avoiding VED, by adding the pre-F to the BARS13 formulation.

We found that the induction of anti-G neutralizing antibodies and protective efficacy were enhanced and VED was further reduced by combining pre-F with BARS13. Importantly, a CD4^+^ T cell epitope on the F2 region of the pre-F was identified that could replace the enhancing role of pre-F protein in the G protein antibody response. Such a combination of an epitopic peptide with the BARS13 selectively enhanced the protective responses against RSV infection induced by the BARS13 vaccine and could lead to a novel strategy for the clinical development of a safe RSV vaccine for man.

## Results

### pre-F facilitates the humoral response induced by the BARS13 vaccine

**Fig. 1:**
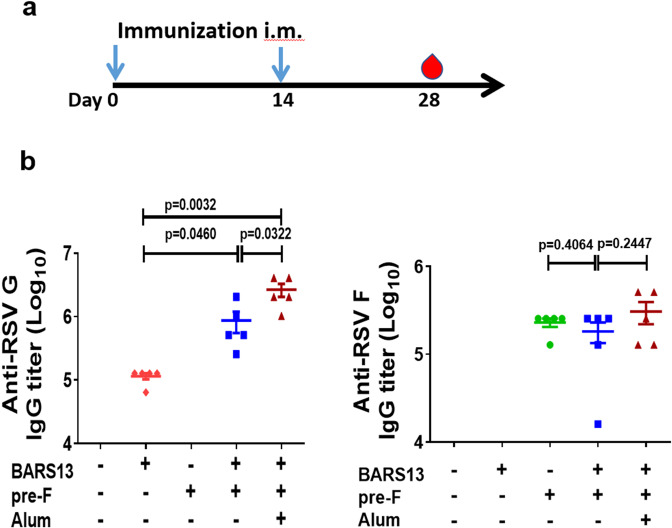
pre-F facilitates the humoral response induced by the BARS13 vaccine. **a** Schematic outline of experimental approach: mice were immunized i.m. twice at days 0 and 14, and blood was collected on day 28. The blue arrows in (**a**) represent time points for immunizations. **b** Titers of anti-G and anti-pre-F IgG were tested on day 28 (*n* = 5 mice per group). These experiments were duplicated at least three times and produced similar results as shown in the Source data. Statistical significance was assessed by two-tailed unpaired Student’s t-test. Data are shown as means ± SEM.

In our previous study, immunization with the BARS13 vaccine protected mice from RSV infection and led to minimal VED. The induction of neutralizing anti-G antibodies and Treg cells by the vaccine was demonstrated to suppress the inflammatory T cells that cause VED. Here, to further improve the effectiveness of the BARS13 vaccine, we formulated a CHO-produced recombinant pre-F protein and the BARS13 vaccine together (BARS13 + pre-F) with or without Alum adjuvant. Mice were immunized i.m. twice with a two-week interval and challenged with RSV-A2 at 2 × 10^6^ pfu intranasally on day 28 (Fig. 1a). Antigen-specific antibodies in serum on days 0 and 28 were sampled. The level of anti-G antibodies induced by the BARS13 was significantly enhanced by the inclusion of pre-F protein when compared with the BARS13 vaccine alone and the inclusion of Alum adjuvant further increased the level of anti-G antibodies (Fig. 1b). The levels of anti-pre-F antibodies were not enhanced by the inclusion of pre-F with BARS13.

Since our previous study showed that the BARS13-induced Tregs played an important role against VED, we asked if the addition of recombinant pre-F into the BARS13 vaccine affected the induction of Tregs. After two immunizations, the levels of Tregs in both spleen and mediastinal lymph nodes (mLN) were higher in the group that received BARS13 + pre-F compared with mice that received pre-F alone or FI-RSV. The Treg levels achieved by BARS13 and BARS13 + pre-F were similar (Supplementary Fig. 1). Thus, the addition of pre-F to the BARS13 vaccine did not affect the induction of Tregs.

### A CD4^+^ T epitope located within the F2 region of pre-F facilitates the anti-G antibody responses

**Fig. 2:**
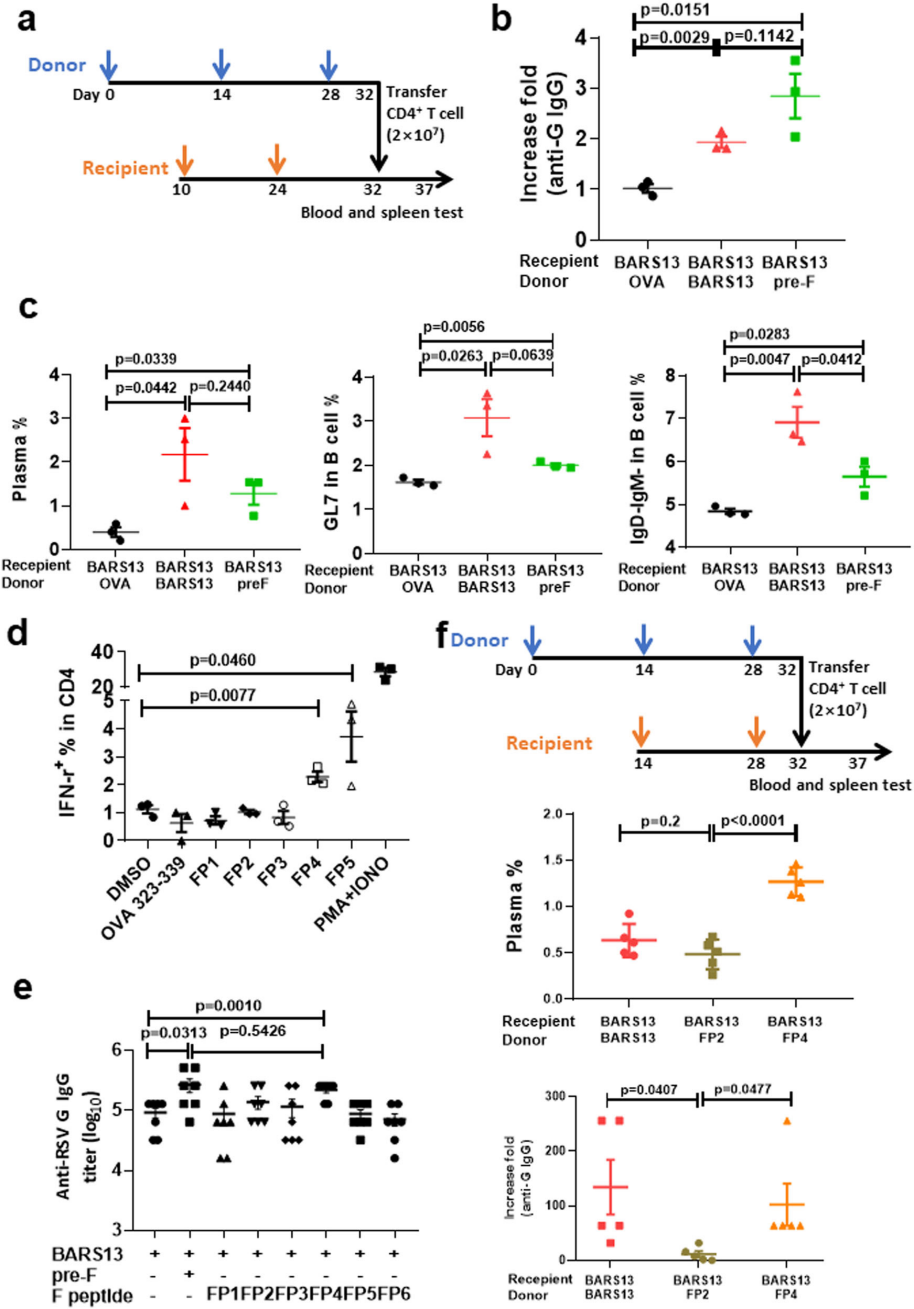
A CD4^+^ T cell epitope within the F2 region of pre-F can enhance anti-G antibody responses. **a** CD4^+^ T cells were obtained by isolation kit from spleens of donor mice 4 days after the third immunization and injected intravenously, 2 × 10^7^ cells per recipient mouse, at 8 days after the second immunization. **b** Antibody against G in the blood of recipient mice was tested at days 31 and 35 and the -fold increase was calculated (*n* = 3 mice per group). **c** Germinal center B cells (B220^+^ GL7^+^) and Ig-switched B cells (B220^+^ IgD^−^ IgM^−^) in the spleens of recipient mice were assayed at day 36 (*n* = 3 mice per group). **d** Percentage of IFN-γ expressing CD4^+^ in splenocytes from mice immunized with pre-F after being stimulated by the predicted peptides in vitro (*n* = 3). **e** Anti-RSV G IgG titer was tested 14 days after the second immunization (*n* = 7 mice per group). **f** CD4^+^ T cells were obtained by isolation kit from the donors’ spleens 4 days after the third immunization and adaptively transferred intravenously into recipients (2 × 10^7^ per mouse) at 4 days after the second immunization (*n* = 5 mice per group). Anti-sera against G protein in recipient mice was tested on day 30 and the -fold increases were calculated. Plasma cells (B220^−^ CD138^+^) in the spleens of recipient mice were assayed at day 37. The blue and yellow arrows in (**a**) and (**f**) represent time points for immunizations. The experiments in (**a**), (**b**), and (**c**) were duplicated and produced similar results as shown in the data set. Statistical significance was assessed by two-tailed unpaired Student’s t-test. Data are shown as means ± SEM.

There were no homologous sequences between the pre-F and the G protein of RSV. Therefore, it appeared unlikely that the pre-F antigen directly stimulated B cells to contribute to the enhancement of anti-G antibodies, but more likely that the pre-F antigen directly stimulated CD4^+^ T cells to provide T help and indirectly facilitate the G antigen activation of B cells. To test the notion, CD4^+^ T cells from donor mice immunized with the BARS13, pre-F, or ovalbumin (OVA) were adoptively transferred into recipient mice that previously had been immunized with the BARS13 (Fig. 2a). Five days after transfer, CD4^+^ T cells from either the pre-F or BARS13-immunized donors had enhanced the anti-G antibody production (Fig. 2b), the levels of plasma cells (B220^−^ CD138^+^), germinal center B cells (B220^+^ GL7^+^), and Ig-switched B cells (B220^+^ IgD^−^ IgM^−^) in recipients compared to the control recipients of T cells from mice immunized with OVA (Fig. 2c). Thus, the T cells activated by the pre-F vaccinations could potentiate the BARS13 vaccine-induced B cells to produce more antibodies against the G antigen by facilitating those B cells to become more mature and developed.

To identify epitope(s) within the pre-F region that caused this potentiation, we employed a web-based analysis tool (Immune Epitope Database analysis, IEDB, http://www.iedb.org/) to predict the stronger CD4^+^ T cell epitopes^36^. We identified six epitopes, one of which overlapped with a previously reported CD4^+^ T cell epitope (Table 1). The ability of the splenocytes from mice immunized with pre-F protein to secrete the cytokine IFN-γ after being stimulated by one of the five novel predicted peptides was tested in vitro. Although both peptides 4 and 5 (FP4 and FP5) stimulated CD4^+^ T cells to secrete a higher level of IFN-γ (Fig. 2d), the level of anti-RSV G antibodies induced by the addition of FP5 was lower than by the addition of FP4 (Fig. 2e). The results suggested that the FP4 peptide could be the primary T cell epitope, located in the F2 region of the pre-F, that potentiated the humoral response to the BARS13 vaccine. To further demonstrate that the FP4 peptide enhanced the humoral response to the BARS13 vaccination through CD4^+^ T cells, we adaptively transferred CD4^+^ T cells from the FP4-immunized mice as donors to the BARS13-immunized recipient mice. The results showed that the donor CD4^+^ T cells that were induced by FP4, not by FP2, could significantly promote the levels of anti-G plasma cells and antibody production (Fig. 2f).

**Table 1:** CD4^+^ T cell epitope in F protein predicted by IEDB.

Name	Complete sequence	Core sequence	Position	Adjusted rank
FP1	TPCWKLHTSPLCTT	PCWKLHTSP	272–285	9.53
FP2	NQSLAFIRKSDELLS	AFIRKSDEL	461–475	8.7
FP3	LSTNKAVVSLSNG	NKAVVSLSN	133–145	3.81
FP4	RTGWYTSVITIELSN	YTSVITIEL	49–63	9.75
FP5	SSSVITSLGAIVSC	SSVITSLGA	364–377	4.68
FP6	VSVLTFKVLDLKN	LTFKVLDLK	143–155	23

Adjusted rank, lower number represents a higher binder.

*FP* pre-F peptide.

**Fig. 3:**
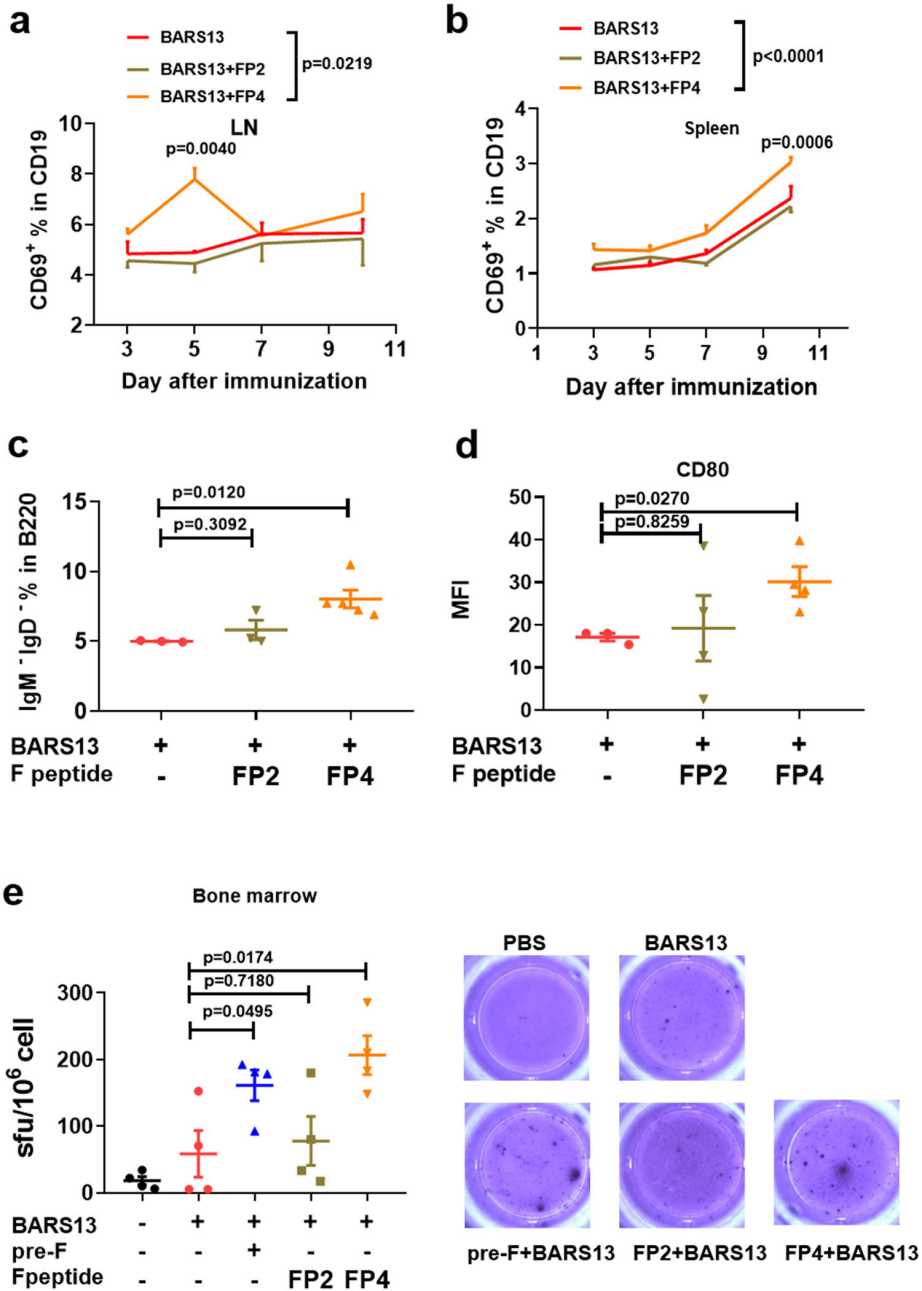
Effects of FP4 on the early activation of B cells induced by the BARS13 vaccine. The level of activated B cells (B220^+^ CD69^+^) in lymph nodes (**a**) and spleens (**b**) collected on days 3, 5, 7, and 10 after a single immunization i.m. (*n* = 3 mice per group). **c** Level of Ig-switched B cells (B220^+^ IgD^−^ IgM^−^) in mouse spleen on day 7 after one immunization; *n* = 3 for BARS13 group, *n* = 5 for BARS13 + FP2 group, *n* = 5 for BARS13 + FP4 group. **d** Mean fluorescence intensity (MFI) of the molecule CD80 on B cells from mouse spleen on day 7 after one immunization; *n* = 3 for the BARS13 group, *n* = 4 for the BARS13 + FP2 group, *n* = 4 for BARS13 + FP4 group. **e** Levels of G-specific ASC were detected by ELISpot after mice were immunized twice on days 0 and 14, and bone marrow cells were obtained on day 35; *n* = 4 mice per group. Statistical significance was assessed by two-way ANOVA with Dunnett’s multiple comparisons test (**a**, **b**) and two-tailed unpaired Student’s t-test (**c**–**e**). Data are shown as mean ± SEM.

To further assess the role of the FP4 in promoting antibody response to BARS13, we first used flow cytometry to assess the frequency of early-activated CD69^+^ B cells in lymph nodes and spleen on days 3, 5, 7, and 10 after a single immunization. As depicted in Fig. 3a, the level of CD19^+^ CD69^+^ cells in the BARS13 + FP4 group was similar to the levels in the BARS13 and BARS13 + FP2 groups at early day 3 but was significantly greater on day 5 before decreasing to a similar level on day 7 in lymph nodes. In the spleen, the level of CD19^+^ CD69^+^ cells of the BARS13 + FP4 group was significantly higher than those of the BARS13 and BARS13 + FP2 groups on day 10 (Fig. 3b). To confirm the early B cell activation, we examined the frequency of Ig-switched B cells (B220^+^ IgD^−^ IgM^−^) on day 7 after the immunization; the proportion of Ig-switched B cells was higher in the BARS13 + FP4 group than in the two control groups, BARS13 and BARS13 + FP2 (Fig. 3c). We also examined the co-stimulating molecule CD80 since it had been indicated as only expressed in activated B cells; CD80 was significantly higher in the BARS13 + FP4 group on day 7 than in the two comparator groups, BARS13 and BARS13 + FP2 (Fig. 3d).

When B cells differentiate into antibody-secreting plasma cells (ASC), some plasma cells circulate back to the bone marrow as long-lived memory B cells. Accordingly, we measured the level of plasma cells in the bone marrow of these immunized mice on day 35 by ELISpot assay. As shown in Fig. 3e and 3f, the frequency of G protein-specific ASCs was significantly increased in the BARS13 + pre-F and BARS13 + FP4 immunized groups above that of the BARS13 group and the BARS13 + FP2 immunization was ineffective. Thus, the FP4 epitope can promote BARS13 to generate long-lived B cells. Since it was previously reported that a few promiscuous CD4^+^ T cell epitopes can facilitate the immune responses for a variety of antigens, we explored if the FP4 epitope peptide has a similar promiscuous stimulation ability. We immunized twice with the FP4 peptide mixed with OVA antigen and tested if the anti-OVA IgG level was augmented on day 28. As depicted in Supplementary Fig. 2, a significantly enhanced level of anti-OVA antibodies was induced in the OVA + FP4 group compared to the OVA alone group. Although we did not test other antigens with the peptide, the result suggests that the FP4 peptide may contain a promiscuous sequence that facilitates antibody responses not only to OVA but possibly also to other antigens.

### FP4 potentiates anti-CCD antibody production

**Fig. 4:**
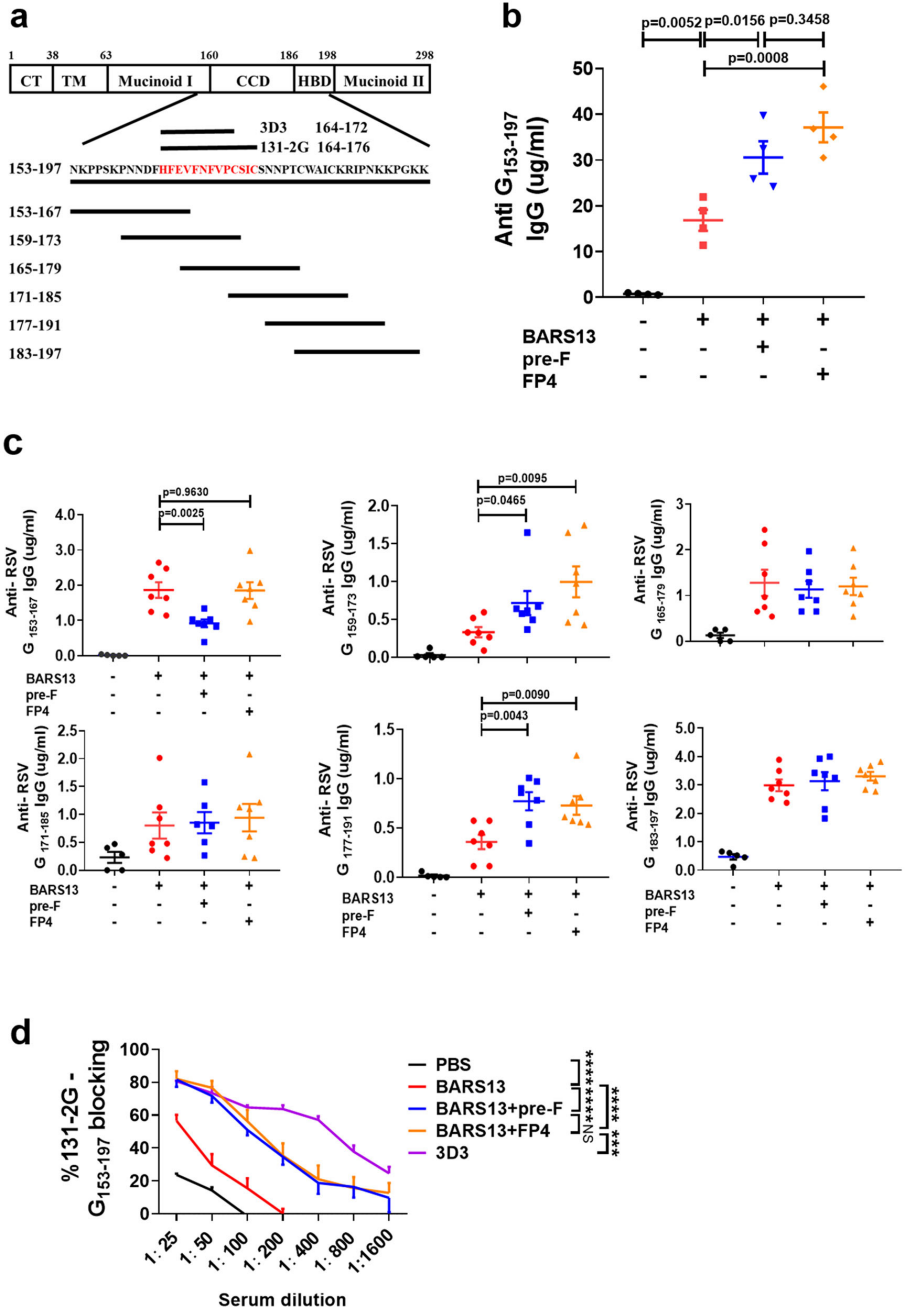
FP4 potentiates anti-CCD antibody production. **a** Diagram of the structure of G protein, location, and sequence of synthesized overlapping peptides of the CCD region. **b** Anti-RSV G_153-197_ antibody was quantified by ELISA using 131-2G as the standard (*n* = 4 mice per group). **c** Anti-RSV G antibodies were tested against overlapping sequences (*n* = 7 mice per group except *n* = 5 for the PBS group). **d** Antibody in serum competes with 131-2G in binding to G_153-197_ in a competitive ELISA (*n* = 4 mice per group). **P = 0.0014, ****P < 0.0001; NS (not significant). These experiments were duplicated and produced similar results as shown in the data set. Statistical significance was assessed by one-way ANOVA with Dunnett’s multiple comparisons test (**b**), two-tailed unpaired Student’s t-test (**c**), and two-way ANOVA with Dunnett’s multiple comparisons test (**d**). Data are shown as means ± SEM.

To determine where on the RSV G protein the enhanced anti-G antibodies were bound, we first generated a long peptide containing the CCD region (Fig. 4a). As depicted in Fig. 4b, the levels of anti-CCD IgG induced by the BARS13 + pre-F and BARS13 + FP4 vaccines were significantly higher than those induced by the BARS13 vaccine. The antibodies against CCD of G protein mainly recognized linear epitopes. To characterize the binding locations, we tested six overlapping peptides covering the CCD region (Fig. 4a) and found that the enhancement in antibody binding occurred mainly to peptides G_159-173_ and G_177-191_ (Fig. 4c).

The sequence of G_177-191_ contains a CX3C motif that serves as the ligand to the host CX3CR1 receptor and G_159-173_ contains the central conserved region (CCR). Since both sequences play a crucial role in early RSV infection, antibodies bound to these sites could neutralize RSV. A competitive ELISA was developed to identify antibodies that recognized G_159-173_ and could compete with a previously known function-neutralizing anti-RSV-G monoclonal antibody, 131-2G^13^. The enhanced anti-serum induced by the BARS13 + pre-F and BARS13 + FP4 vaccines more effectively competed with 131-2G to bind to the CCR when compared with the BARS13-induced anti-serum. Another anti-G monoclonal antibody, 3D3, that bound to a similar site on 131-2G, was used as the positive competitor control (Fig. 4d). Thus, the pre-F protein and FP4 changed the quality and composition of the anti-G antibodies more toward the CCD region.

### FP4 potentiates the protective efficacy of the BARS13 vaccine against RSV

**Fig. 5:**
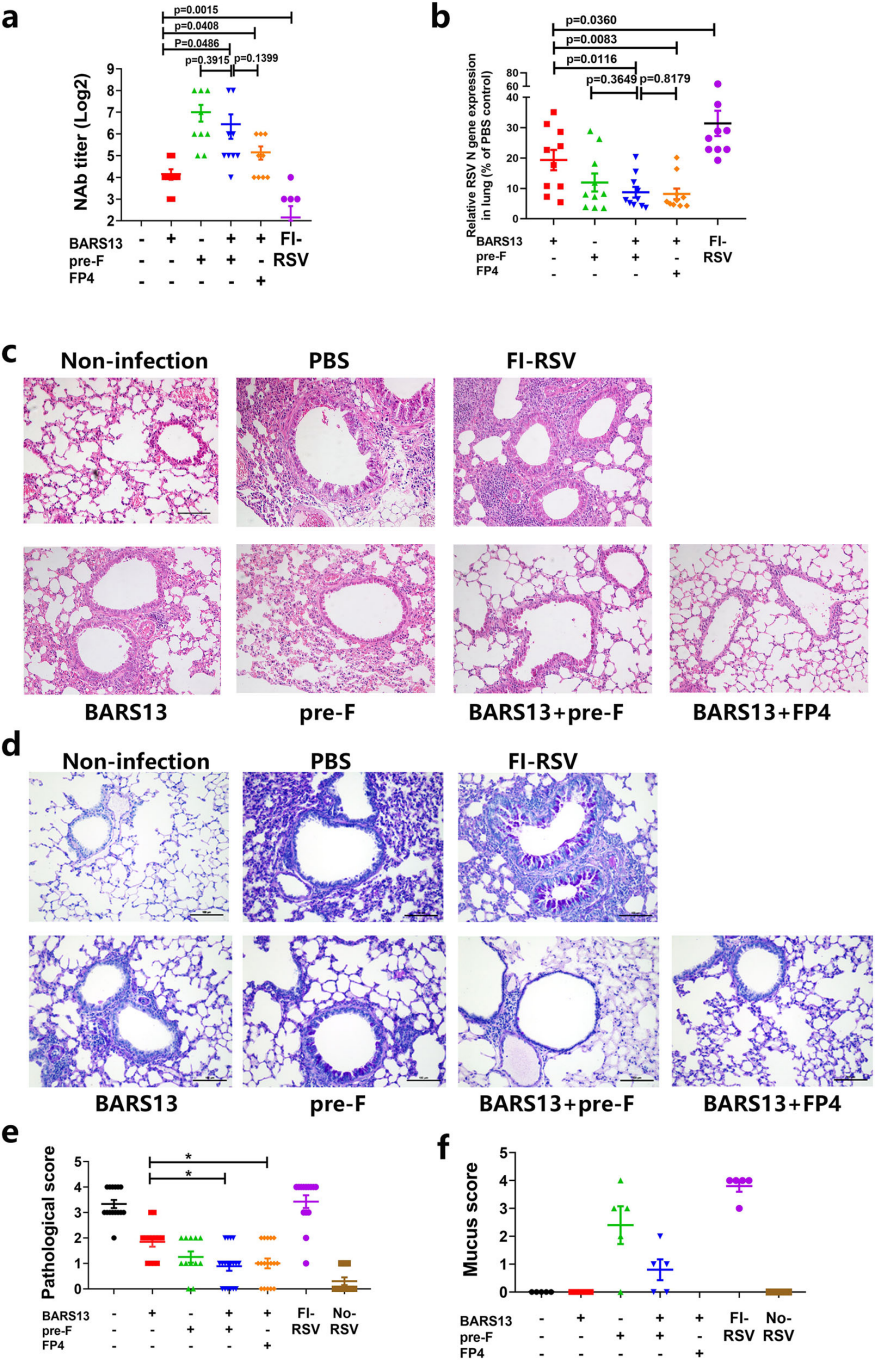
FP4 potentiates the protective efficacy of the BARS13 vaccine against RSV. Mice were immunized twice on days 0 and 14 and were challenged on day 28. **a** NAb was tested at day 33 (*n* = 9 mice per group). **b** Virus loads in the lung were tested on day 33 (*n* = 10 mice per group). **c** Representative lung section made at 5 dpi and stained with HE (*n* = 8 mice per group). **d** Representative lung section made at 5 dpi and stained with PAS (*n* = 8 mice per group). **e**, **f** Score for the sections after HE (**e**) and PAS staining (**f**) (*n* = 4 mice per group). NAb, neutralizing antibody. Scale bar in (**c**) and (**d**) represents 100 μm. These experiments were duplicated three times and produced similar results as shown in the data set. Statistical significance was assessed by a two-tailed unpaired Student’s t-test. Data are shown as means ± SEM.

To examine if the BARS13 + FP4 could also induce enhanced protective efficacy against RSV infection, 6-week-old female mice were immunized with the various vaccines, including the FI-RSV as a positive control for detection of VED, then challenged with 2 × 10^6^ pfu of RSV-A2 14 days after the last immunization. Five days after challenge infection, the levels of neutralizing antibodies in mice immunized with the pre-F, BARS13 + pre-F, or BARS13 + FP4 were similar, but significantly higher than in the BARS13-immunized, whereas FI-RSV induced the lowest level (Fig. 5a). The virus loads in lungs from the BARS13 + pre-F and BARS13 + FP4 groups were significantly reduced compared to the BARS13 group, whereas FI-RSV reduced the viral load the least (Fig. 5b). The histopathology of the infected lungs showed by H&E staining that the order of degree of inflammatory cell infiltration, ranked from least to greatest, was BARS13 + pre-F = BARS13 + FP4 < pre-F < BARS13 < FI-RSV vaccine (Fig. 5c, e). PAS staining revealed that the amount of mucus secretion in the lung, ranked from the least to greatest, was BARS13 + FP4 = BARS13 < BARS13 + pre-F < pre-F < FI-RSV (Fig. 5d, f). Thus, the T cell epitope FP4 could potentiate the BARS13 vaccine-induced anti-RSV infection and greatly reduce the lung pathogenesis known as VED, which has become a major obstacle to the RSV vaccine development.

## Discussion

There was a disastrous failure of FI-RSV vaccines developed more than 50 years ago due to enhanced lung inflammatory reactions during subsequent RSV infections, which later became known as vaccine-enhanced disease (VED) or enhanced respiratory disease (ERD). Key reasons for the enhanced disease include induction of low neutralizing antibody responses and Th2-biased T cell responses after FI-RSV, but the mechanisms are not fully understood^37^. The risk of exacerbating RSV infections has held back the development of RSV vaccines.

The F and G proteins on the surface of RSV are the major targets for neutralizing antibodies and are the favored immunogens for RSV vaccine development. In this study, we demonstrated that the addition of pre-F to the BARS13 formulation increased the neutralizing antibody response and still reduced the risk of VED in the mouse model (Fig. 5). Our observation that the addition of pre-F could facilitate the induction by BARS13 of a significantly higher level of anti-G antibody production led us to discover a T cell epitope (FP4) located within the F2 region of the pre-F protein and serving as a T helper epitope to potentiate anti-G antibodies. The presence of the FP4 epitope within pre-F not only facilitated anti-G antibody production but also enhanced anti-RSV neutralizing function and resulted in protective responses against the RSV infection in the animal model. This enhancement could not be due to the induction of anti-FP4 antibodies since no such antibody was induced by this peptide when we immunized animals with it (Supplementary Fig. 3). The enhanced antibodies were raised against the most conserved sequence in the G antigen at aa159–197, where the CX3CR1 receptor binding domain is located. Notably, the presence of increased anti-G antibodies induced neither VED nor increased mucous secretion with subsequent RSV challenge (Fig. 5).

Previous studies have reported that the addition of the pre-fusion F protein could enhance anti-G antibodies more than the post-fusion F protein^38^; our present study demonstrated that such enhancement was due to the induction of the F-specific T cells. The better enhancement by pre-F may be due to the FP4 located close to the antibody epitope Φ on pre-F, which could favor the presentation of FP4 by B cells to generate the stronger FP4-specific T cell response. Although the sequence of FP4 is present in both forms of F protein, MHC-II of antigen-presenting cells only recognize the linear sequence of peptide epitopes regardless of conformations. When a core sequence of the peptide fits into the shallow groove of the MHC-II antigen-binding site, flanking residues of the peptide can greatly affect its binding affinity with the MHC-II molecule. For example, the epitope sequence of FP4_49-63_ contains the same core sequence, “YTSVITIEL”, at F_53-61_ of the F2 region of pre-F, and it was rated to have stronger binding to MHC-II than the previously discovered peptide F_51-65_. This suggests that the extra flanking amino acid residues indeed contribute to its affinity^39^. Several previous studies documented that some T helper cell epitopes, such as the PADRE peptide and Tetanus toxoid that act as or contain promiscuous epitopes, could be used to enhance the antibody responses both to antitumor vaccines and to a recombinant SARS-CoV-2 RBD vaccine^40–43^. Since B cell activation and enhancement mainly depends on the interaction of Tfh cells in the germinal center, we speculate that the FP4 epitope might also stimulate the Tfh cells to bring about those enhancements. In addition, a previous study has reported that peptide F_49-66_ can act as a CD4^+^ T epitope, stimulating human PBMC to secrete IFN-γ, indicating that this FP4 epitope can stimulate T cells from both mice and humans^44^. If the FP4 epitope has such potential, in addition to promiscuous stimulation, it might find application in other vaccine designs as a B cell potentiator.

The central conserved region of the G protein contains a sequence motif similar to one in chemokine CX3C that can favor RSV infection; it triggers excessive inflammation by interaction with CX3CR1 on lung epithelial cells and immunocytes, including some T cells, B cells, and monocytes^10,19–22,24,25^. Antibodies against the CCD in CX3C could block the CX3C-CX3CR1 reaction and prevent RSV infectivity and breakthrough^9,15,45^, and result in the reduction of CX3CR1^+^ T cell trafficking to the lung and the elimination of the VED^46^. Therefore, the induction of antibodies against a CCD, such as the one in CX3C, is a vital point for the G protein-based RSV vaccine design. In this study, we showed that the BARS13 vaccine formulated with the pre-F or FP4 induced higher levels of anti-CCD antibodies than the BARS13 vaccine alone and enhanced protection against the RSV infection with less VED. This indicated that the enhanced anti-CCD antibodies may inhibit infection by blocking CX3C-CX3CR1 binding and could also contribute to less inflammation.

In this study, the pre-F protein was found to induce some degree of mucus production, which was consistent with previous reports^47,48^. This mucus induction was not less in the BARS13 + pre-F group, whereas there was significantly less mucus induction in the BARS13 + FP4 immunized group. This phenomenon is currently difficult to explain and needs further investigation.

In conclusion, we found that the FP4 sequence of the F protein contains a CD4^+^ T cell epitope within the F2 region which can enhance antibody responses against the CCD and CX3C regions of the RSV G protein. Such enhancement led to a higher level of neutralizing antibodies against RSV and protected mice from RSV challenge while minimizing VED. The combined effects indicate a novel strategy for developing a safe and effective recombinant G protein-based RSV vaccine.

## Methods

### Mice

Female 6- to 10-wk-old BALB/c mice used for all experiments were purchased from Shanghai Jiesijie Laboratory Animal Co., Ltd. (Shanghai, China) and grew under specific pathogen-free conditions at Fudan University. All animal experiments were approved by the Committee of Experimental Animals of Shanghai Medical College (SHMC), and carried out in compliance with the ARRIVE guidelines. All mice were sacrificed under euthanasia with isoflurane treatment at the end of experiments.

### Cells, proteins, peptides, and virus

Vero was purchased from ATCC (Manassas, VA, USA) and cultured in DMEM medium (Gibco) supplemented with 10% FBS (Gibco) and 1% antibiotics (Gibco), respectively.

*E. coli*-produced recombinant G protein, CHO cell-produced pre-F, and CsA adjuvant were kindly provided by Advaccine Biopharmaceutics (Suzhou) Co. Inc (Suzhou, China). Ovalbumin (OVA) was purchased from Sigma (Boston, USA). The chimeric 131-2G was composed of mouse variable domains and rabbit constant domain (IgG1Fc) and was expressed by Biointron Biotech Co., Ltd (Shanghai, China). The 3D3, antibody against RSV G_164-172_, was kindly provided by Dr. Bing Sun (Shanghai Institute of Biochemistry and Cell Biology, Chinese Academy of Sciences, China). All peptides used in this study were synthesized by Genscript (Nanjing, China).

RSV-A2 was purchased from ATCC and grown in Vero cells. The virus titer was determined in Vero cells, adjusted to half of the tissue infection dose (TCID_50_), and converted to PFUs according to the following formula: PFUs = 0.7 × TCID_50_.

### Vaccine preparations, immunization, and RSV challenge

Four vaccines were compared: BARS13 (10 μg G protein + 10 μg CsA), pre-F (10 μg), BARS13 + pre-F (10 μg G protein + 10 μg CsA + 10 μg pre-F) with or without 100 μg Aluminum hydroxide adjuvant (Invivogen, CA, USA), BARS13 + pre-F peptide (10 μg G protein + 10 μg CsA10 μg + 10 μg pre-F peptide) and FI-RSV. Control FI-RSV was prepared by adding 25 μl formalin to 100 ml of 10^6^ pfu/ml RSV and incubated at 37 °C for 72 h. After centrifuging at 50,000 × *g* for 1 h at 4 °C, pellets were re-suspended with 4 ml DMEM medium and mixed with a final concentration of 4 mg/ml Aluminum hydroxide adjuvant overnight. After centrifugation again at 3000 rpm for 30 min at 4 °C, the precipitates were re-suspended with 1 ml DMEM medium, aliquoted for the 4 °C storage before use^49^. The mice were immunized i.m. on days 0 and 14, and blood samples were taken on days 0, 14, and 26. On day 28, the mice were infected with RSV-A2 (2 × 10^6^ pfu) via the nasal route under anesthesia. Five days later, the mice were killed and lungs were taken for measurement of the viral loads and pathological evaluation.

Immunization with OVA was done i.m. at days 0 and 14 with 10 μg OVA, 10 μg OVA + 10 μg FP4, or 10 μg OVA + 100 μg aluminum hydroxide adjuvant, and blood samples were collected for anti-OVA IgG detection at day 28.

### Assay of antibody

Serum samples were collected before the challenge on day 26 and after the challenge on day 33. 96-well plates (Costar, NA, USA) were coated with 2 μg/mL G protein or 1 μg/mL pre-F protein at 4 °C overnight, then blocked with 3% BSA in PBST (0.05% Tween 20 in PBS) and serial 2-fold diluted serum was placed into the plate for 1 h at 37 °C. After washing with PBST, the plate was incubated with HRP-conjugated goat anti-mouse IgG (1:4000) (Southern Biotech, Birmingham, AL, USA) and developed by adding TMB for 5 min. The colorizing reaction was stopped by adding 50 μL of 2 M H_2_SO_4_ and absorbance was measured at 450/620 nm by an ELISA plate reader (Bio-Rad, CA, USA). The mean OD (optical density) of sera from mice immunized with PBS was multiplied by 2.1 to define the cutoff point for positivity.

The neutralization assay was conducted by following steps: a 65 μl heat-inactivated serum was a two-fold serial diluted, and each mixed with 35 μl RSV-A2 (1 × 10^4^ pfu) for 2 h at 4 °C then added to Vero cells in a 96-well plate containing 2 × 10^4^/well. After another 2 h, 100 μl DMEM containing 4% FBS was added to the plates. After five days, cells were fixed with 100 μl of 80% cold acetone in PBS after the medium was removed and then blocked with 200 μl PBST containing 5% BSA. One hundred microliters of goat anti-RSV antibodies at a dilution of 1:2000 (Meridian Life Science, Tennessee, USA) was added to the plate for 1 h at 37 °C. The plates were washed with PBST and then incubated with 100 μl donkey anti-goat IgG-HRP at a dilution of 1:4000 (Genscript, A00178) for 1 h at 37 °C. The plate was developed with 100 μl TMB, stopped with 50 μl H_2_SO_4_, and ODs were read with the microplate reader.

For the detection of the OVA-induced antibody, the 96-well plates were coated with 5 μg/mL of OVA in carbonate-bicarbonate buffer (pH 9.6) at 4 °C overnight and then blocked with 3% BSA in PBST (0.05% Tween 20 in PBS). The serum samples at 1:1000 dilution in the 1% BSA were added and incubated for 1 h at 37 °C. After washing with PBST, the plates were incubated with HRP-conjugated goat anti-mouse IgG at 1:4000 dilution (Southern Biotech), color was developed with TMB and the reaction was stopped by adding H_2_SO_4_. ODs were read at 450/620 nm with an ELISA plate reader.

### Lung pathology

Lung samples of mice were fixed in 4% PFA 24 h and then embedded in paraffin. Transverse sections were cut and stained with hematoxylin and eosin (HE) or Periodic Acid-Schiff (PAS). Images of staining were recorded and the degree of lung inflammation and damage was evaluated by scoring the infiltration of inflammatory cells and mucus secretions: 0—normal; 1—minimal; 2—slight; 3—moderate; 4—severe.

### Viral loads analysis

Five days after the challenge, mice were anesthetized by isoflurane and their blood samples were collected before killing. Lung tissues were removed and weighed, followed by tissue homogenization for RNA extraction (Omega Bio-Tek, Georgia, USA). Copies of the RSV N gene in samples were determined by qPCR (Yeasen, Shanghai, China), in which a plasmid containing the RSV N gene sequence was used as the positive control. The primers used were as follows: forward primer: TACGGTGGGGAGTCTTAGCA; reverse primer: CACCACCCAATTTTTGGGCA (Genscript). The viral loads were expressed as a percentage of those found in the animal group administrated with PBS and challenged.

### Competitive ELISA

Biotin-labeled CCD (RSV G_153-197_) was coated with 0.125 μg/mL in a 96-well plate at 4 °C overnight. After the plates were blocked with 3% BSA in PBST, the serially diluted sera were added and incubated at 37 °C for 1 h. The serially diluted 3D3 monoclonal antibody was used as a positive control. The 131-2G at 0.25 μg/mL (Biointron Biotech Co., Ltd, Shanghai, China) was added to the plates and reacted at 37 °C. Simultaneously, 131-2G at a range of dilutions was used for standard curves. After 1 h, the goat anti-rabbit IgG-HRP at 1:4000 dilution (Invitrogen, CA, USA) was used to assess the 131-2G concentration bound to biotin-labeled CCD. The blocking rate was calculated according to the following formula: blocking ratio = (1 – experimental group/blank group) × 100%.

### Semi-quantitative ELISA

The 96-well plates were coated with 5 μg/mL of the CCD peptide or customized synthetic peptides (RSV G_153-167_, G_159-173_, G_165-179_, G_171-185_, G_177-191_, G_183-197_) at 4 °C overnight and blocked with 3% BSA in PBST. Serum samples were diluted 1:50, added to the wells, and incubated at 37 °C for 1 h; serial diluted 131-2G (Sigma, MAB858-2) was used as the positive control. After washes, the goat anti-mouse IgG HRP at 1:4000 dilution (Southern Biotech) was used to react with the bound mouse antibodies. The concentration of mouse anti-peptide antibodies was estimated from the standard curve prepared with 131-2G.

### Isolation and transfer of CD4^+^ T cell

A Murine CD4 T Cell Isolation Kit (Biolegend, CA, USA) was used to isolate CD4^+^ T cells from the spleens of donors that had been immunized with the BARS13, or pre-F, or pre-F peptide or were naïve. The cells were diluted to 2 × 10^7^ cells in 200 μL PBS and injected intravenously into each recipient mouse after immunization with BARS13. Antibodies against the G antigen in the sera of recipient mice were quantified by ELISA one day before the transfer and 5 days after the transfer. Plasma cells (B220^−^ CD138^+^), germinal center B cells (B220^+^ GL7^+^), and Ig-switched B cells (B220^+^ IgD^−^ IgM^−^) in spleens of recipient mice were analyzed 5 days after the transfer.

### CD4^+^ T cell epitope prediction

The prediction of CD4^+^ T cell epitopes of the pre-F protein was performed using Immune Epitope Database analysis (www.IEDB.org)^36,50^. The IEDB web server calculated the peptide binding to the alleles H2-IA^d^ and H2-IE^d^ MHC class II molecules by using the IEDB-recommended 2.22 method. Ten sequences were selected according to the core sequence with the strongest binding ability. The final sequences were determined by excluding some that had proven to be invalid in the previous literature or were ruled out as epitopes based on protein structure.

### G-specific ASC (antibody secreting cells) detection by ELIspot

PVDF membrane 96-well plates (Millipore, Boston, USA) were pre-wetted with 35% ethanol, followed by three washes with PBS, and coated with 20 μg/mL of the G protein overnight at 4 °C. Plates were washed and blocked with RPMI1640 containing 10% heat-inactivated FBS for 1 h. Single-cell suspensions were prepared from bone marrows of twice-immunized mice on day 35 and counted after the lysis of erythrocytes. After removing the blocking solution, 2.5 × 10^5^ cells were added to each well of the 96-well plates and incubated in a CO_2_ incubator for 20 h. Plates were washed after removing the cells and incubated with anti-mouse IgG detection antibody at 1 μg/mL for 2 h at room temperature according to the Mouse IgG ELIspot basic kit (Dakewei Biotech Co., Ltd, Shenzhen, China). Spots were detected by an AID ELIspot reader (AID, Germany) and spot-forming units (SFU) per million cells were calculated.

### Flow cytometry

After erythrocyte lysis, single-cell suspensions were prepared from spleens or lymph nodes of immunized mice. Cells were counted and stained with Fixable Viability Dye eFluor™ 780 (eBioscience, OR, USA) at 1:2000 dilution in PBS containing 2% FBS to remove dead cells before antibody staining. Cells were incubated in PBS (containing 2% FBS) with the following anti-mouse antibodies (all at 1:200 dilution): anti-CD4-PECy7 (eBioscience, 25-0041-82), anti-B220-Pacific Blue (Biolegend, 103227), anti-GL-7-PE (Biolegend, 144608), anti-CD138-APC (Biolegend, 142506), anti-IgM-APC-Cy7 (Biolegend, B194508), anti-IgD-FITC (Biolegend, B147607), anti-CD69-PECy7 (Biolegend, 25-0691-81), and anti-CD80-BV605 (eBioscience, 63-0801-82) for 15 min at room temperature for analyzing the germinal center B cells (B220^+^ GL7^+^), plasma cells (B220^−^ CD138^+^), Ig-switched B cells (B220^+^ IgM^−^ IgD^−^) and MFI of CD80 on the activated B cells. For Foxp3 analysis, cells were fixed and permeabilized with a Foxp3/Transcription Factor Staining Buffer Set (eBioscience, 00-5523-00) following the manufacturer’s protocol. After fixation, intracellular Foxp3 was labeled by anti-Foxp3-APC (eBioscience, 48-5773-80) at 1:100 dilution in a diluted permeabilization buffer for 1 h at room temperature.

For the detection of intracellular cytokines, the splenocytes from mice immunized with pre-F protein were stimulated by peptides at 40 μg/mL for 6 h and PMA (50 ng/mL)/Ionomycin (1 μg/mL) (BD, CA, USA) as the positive control, with blocking by 1 μg/mL of Brefeldin A (BD). Anti-IFN-γ-BV421 (Biolegend, 505830) was incubated (at 1:100 dilution in permeabilization buffer) at room temperature for 1 h after surface staining with anti-CD4-PECy7 (eBioscience, 25-0041-82) and fixation with formaldehyde at room temperature for 8 min. Samples were subjected to LSRFortessa flow cytometry (BD) and analyzed by FlowJo software (Tree Star, Ashland, OR, USA).

### Statistical analysis

Statistical analysis was done using GraphPad Prism 8.0 (GraphPad Software, San Diego, CA, USA) and results are presented as mean ± standard error of the mean (SEM). The exact sample size (*n*) for each experimental group is indicated in the figure legends. Differences between two groups were analyzed with unpaired t-tests. One-way or two-way analysis of variance (ANOVA) was used followed by Dunnett’s multiple comparisons test for comparison among groups. *P* values < 0.05 were considered statistically significant.

### Reporting summary

Further information on research design is available in the Nature Research Reporting Summary linked to this article.

## Supplementary information

Supplementary Figures

DATA SET presented in the Manuscript

REPORTING SUMMARY

## Data Availability

The data supporting this study’s findings are available from the corresponding author upon reasonable request.
