# Supplementary material for: RSV pre-fusion F protein enhances the G protein antibody and anti-infectious responses
Source: NPJ Vaccines. 2022 Dec 19;7:168. doi: 10.1038/s41541-022-00591-w (PMC9762623; doi:10.1038/s41541-022-00591-w)
Supplement: Supplementary file 1 — Supplementary Figures [file 41541_2022_591_MOESM1_ESM.pdf]

SUPPLEMENTARY FIGURES

Supplementary Figure 1. Level of Tregs in spleen and mLN (mediastinal lymph nodes) of immunized mice

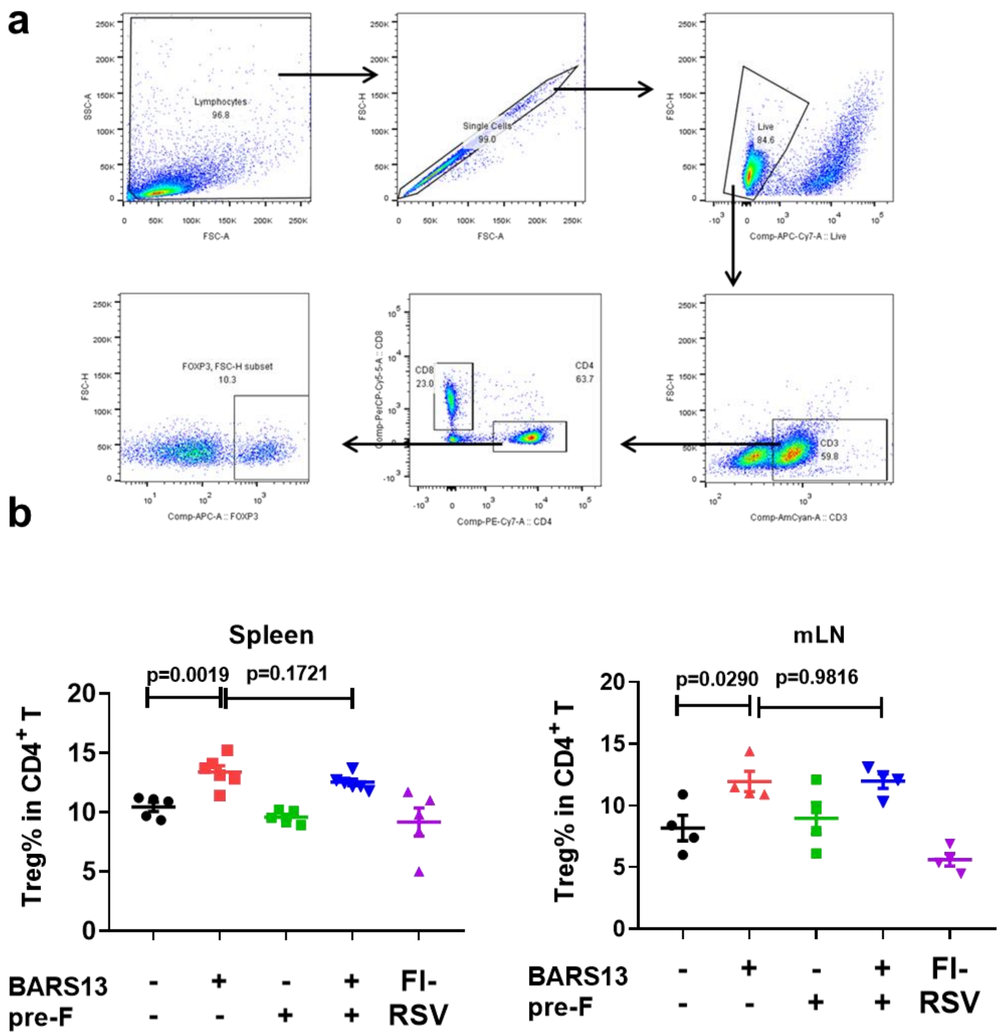

The mice were immunized with PBS, BARS13, pre-F, BARS13+pre-F, and FI-RSV vaccines on days 0 and 14. Percentage of Tregs over total CD4 T cells isolated from spleens and mLNs were analyzed by flow cytometry 5 days after the RSV challenge. Statistical significance is indicated: * $p < 0.05$, ** $p < 0.01$ by unpaired Student's t-test. Data are shown as means ± SEM.

Supplementary Figure 2. Effect of the FP4 on antibody responses induced by OVA

immunization

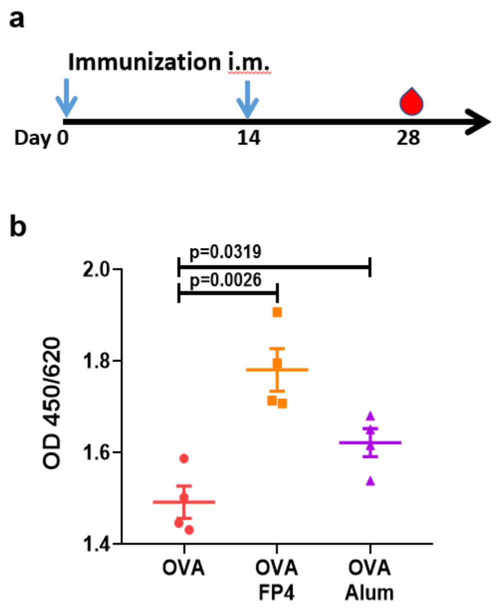

Levels of anti-OVA antibodies in sera were examined by ELISA on day 28 after two i.m. immunizations on days 0 and 14 with 10 µg of OVA, 10 µg OVA+10 µg FP4, 10 µg OVA+100 µg Alum adjuvant. Statistical significance is indicated: * $p < 0.05$, ** $p < 0.01$ by unpaired Student's t-test. Data are shown as means ± SEM.

Supplementary Figure 3. Detection of anti-pre-F IgG induced by FP4

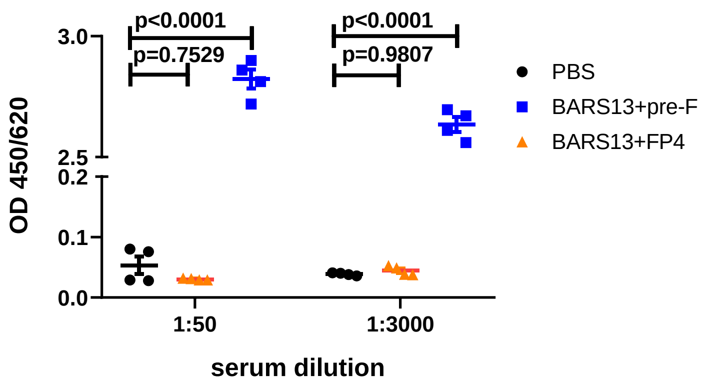

Levels of anti-pre-F antibodies in sera were examined by ELISA on day 28 after two i.m. immunizations with BARS13+pre-F or BARS13+FP4 on days 0 and 14. PBS served as a negative control. Statistical significance was assessed by two-way ANOVA with Dunnett's multiple comparisons test. Data are shown as means ± SEM.

Supplementary Figure 4. Gate strategy for the detection of B cell

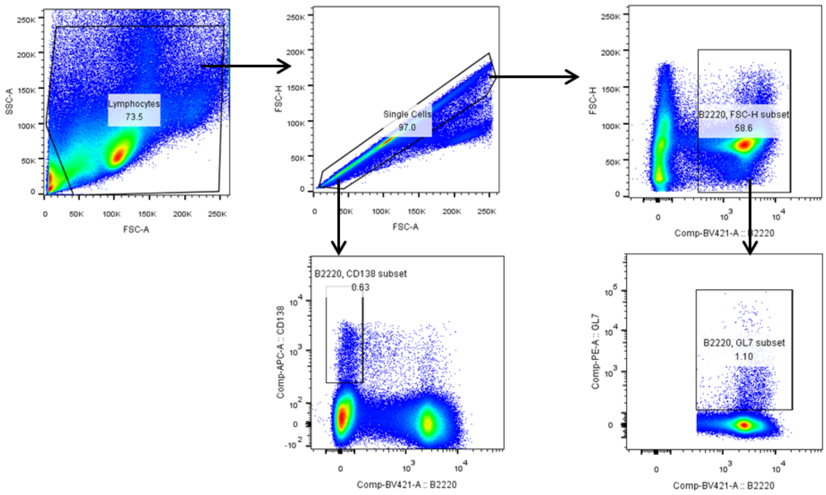

B cells was first identified by forward and side scatter profiles, then forward scatter area versus height was used to select single cells. Then B220$^-$ CD138$^+$ cell was identified. Other B cells were identified by detecting their markers on B220+ cells.
