# Supplementary material for: RSV pre-fusion F protein enhances the G protein antibody and anti-infectious responses
Source: NPJ Vaccines. 2022 Dec 19;7:168. doi: 10.1038/s41541-022-00591-w (PMC9762623; doi:10.1038/s41541-022-00591-w)
Supplement: Supplementary file 2 — DATA SET presented in the Manuscript [file 41541_2022_591_MOESM2_ESM.docx]

**DATA SET presented in the Manuscript**

**Figure 1. pre-F facilitates the humoral response induced by the BARS13 vaccine**

**
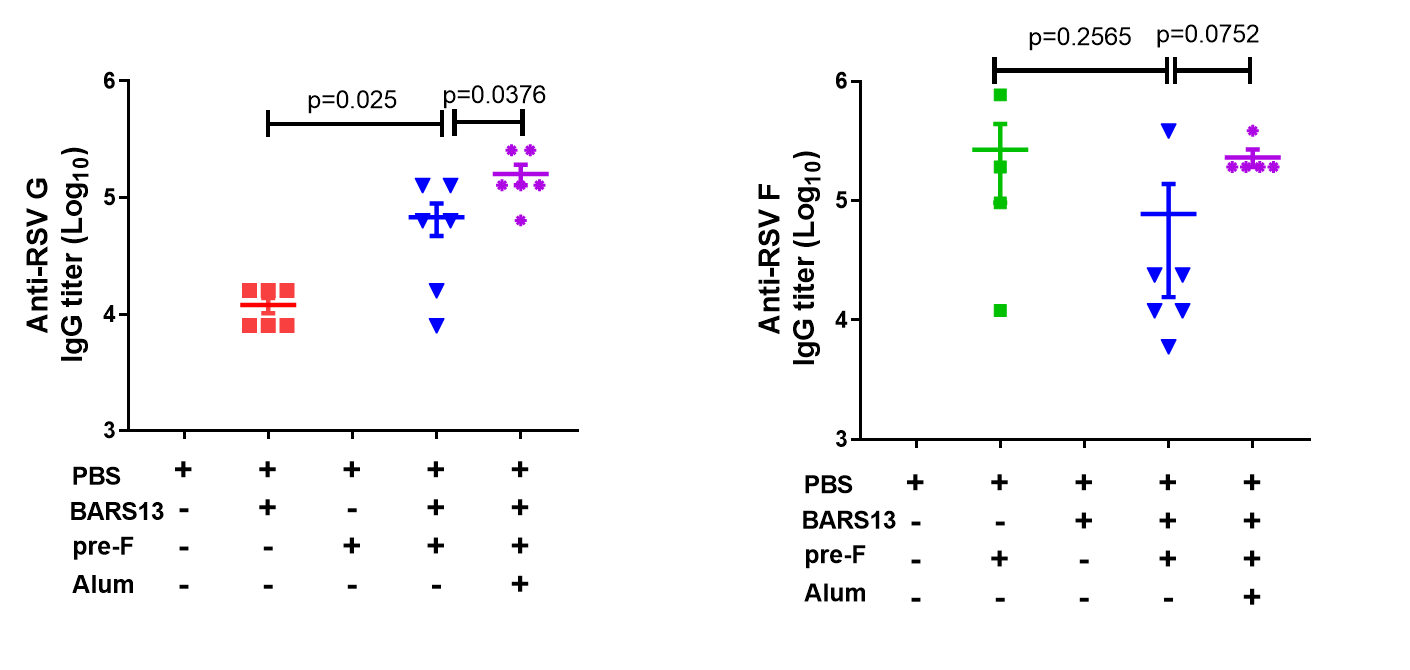
**

Titers of anti-G and anti-pre-F IgG were tested on day 28. Statistical significance was assessed by two-tailed unpaired Student’s t-test. Data are shown as means ± SEM.

**Figure 2. A CD4^+^ T cell epitope within the F2 region of pre-F can enhance anti-G antibody responses**


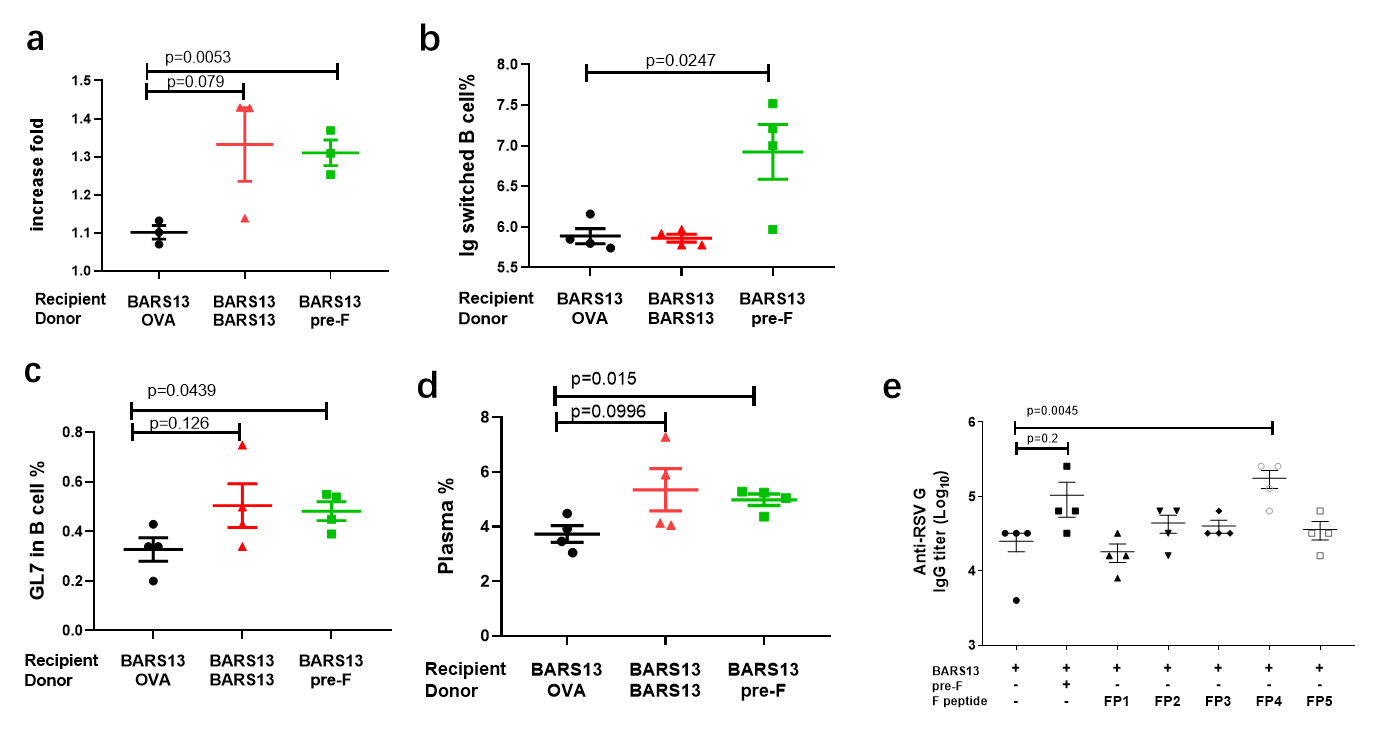


**a,** Antibody against G in the blood of recipient mice was tested at days 31 and 35 and the fold-increase was calculated. **b,** Ig-switched B cells (B220^+^ IgD^-^ IgM^-^), **c,** Germinal center B cells (B220^+^ GL7^+^), and **d,** plasma cells (B220^-^ CD138^+^) in the spleen of recipient mice were tested at day 36. **e,** Anti-RSV G IgG titer was tested 14 days after the second immunization. Statistical significance was assessed by two-tailed unpaired Student’s t-test. Data are shown as ± SEM.

**Figure 3. FP4 potentiates anti-CCD antibody production**


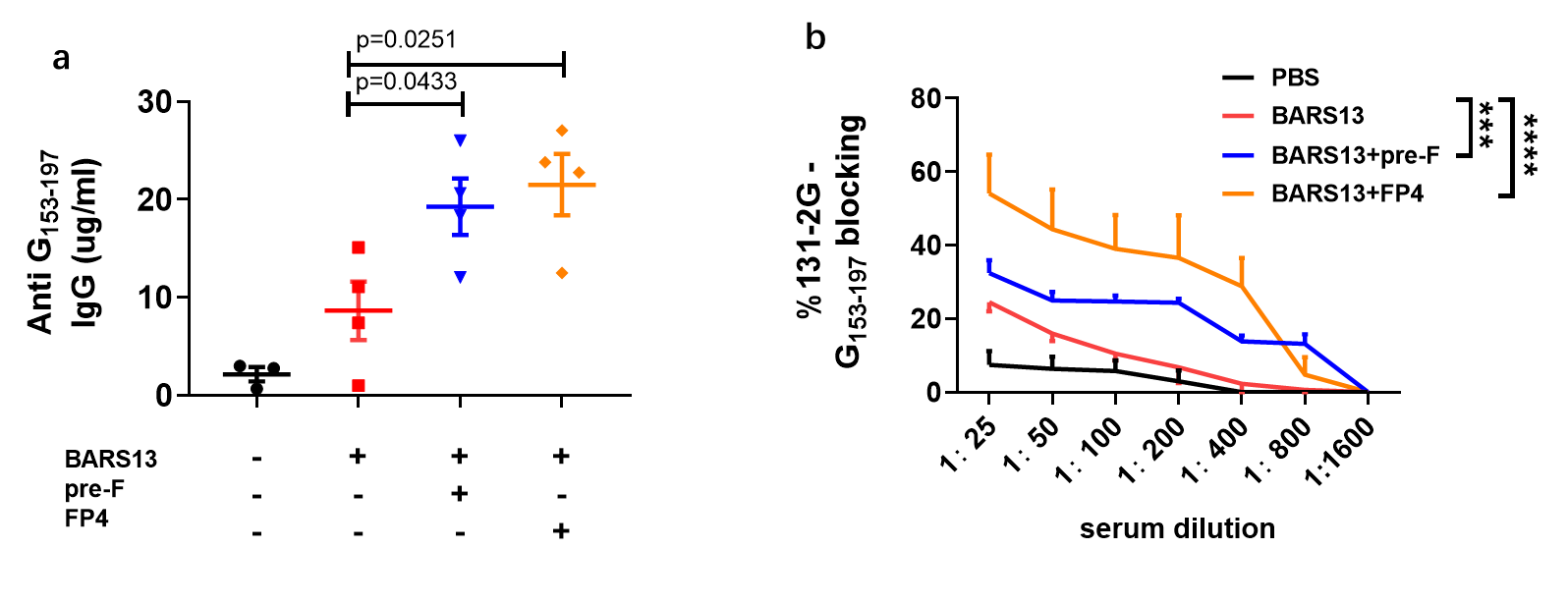


**a,** Anti-RSV G antibodies from animals immunized with indicated vaccines were tested against the G_153-197_ peptide. **b,** Anti-G antisera compete with the 131-2G in binding to G_153-197_ in a competitive ELISA. Statistical significance was assessed by one-way ANOVA with Dunnett's multiple comparisons test (b), two-tailed unpaired Student’s t-test (c) and two-way ANOVA with Dunnett's multiple comparisons test (d). Data are shown as means ± SEM.

**Figure 4. FP4 potentiates the protective efficacy of the BARS13 vaccine against RSV**


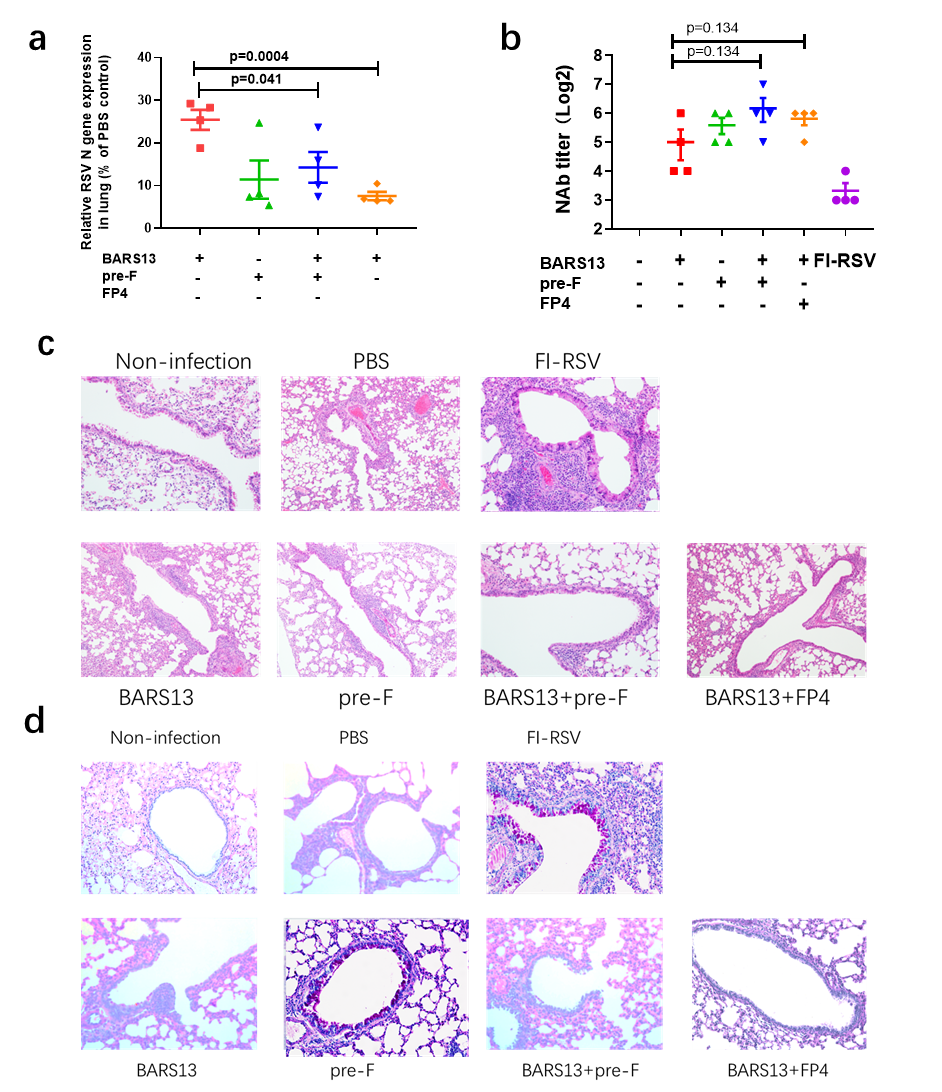


Mice were immunized twice on days 0 and 14 and were challenged on day 28. **a,** NAb was tested at day 33. **b,** Virus loads in the lung were tested on day 33. **c,** Representative lung section made at 5 dpi and stained with HE. **d,** Representative lung section made at 5 dpi and stained with PAS. NAb, neutralizing antibody. Statistical significance was assessed by two-tailed unpaired Student’s t-test. Data are shown as means ± SEM.
