# Supplementary material for: RSV pre-fusion F protein enhances the G protein antibody and anti-infectious responses
Source: NPJ Vaccines. 2022 Dec 19;7:168. doi: 10.1038/s41541-022-00591-w (PMC9762623; doi:10.1038/s41541-022-00591-w)
Supplement: Supplementary file 3 — REPORTING SUMMARY [file 41541_2022_591_MOESM3_ESM.pdf]

nature portfolio

Corresponding author(s): Professor Bin Wang

Last updated by author(s): Nov 1, 2022

Reporting Summary

Nature Portfolio wishes to improve the reproducibility of the work that we publish. This form provides structure for consistency and transparency in reporting. For further information on Nature Portfolio policies, see our Editorial Policies and the Editorial Policy Checklist.

Statistics

For all statistical analyses, confirm that the following items are present in the figure legend, table legend, main text, or Methods section.

n/a	Confirmed	
☐	☒	The exact sample size (n) for each experimental group/condition, given as a discrete number and unit of measurement
☐	☒	A statement on whether measurements were taken from distinct samples or whether the same sample was measured repeatedly
☐	☒	The statistical test(s) used AND whether they are one- or two-sided<br>*Only common tests should be described solely by name; describe more complex techniques in the Methods section.*
☐	☒	A description of all covariates tested
☐	☒	A description of any assumptions or corrections, such as tests of normality and adjustment for multiple comparisons
☐	☒	A full description of the statistical parameters including central tendency (e.g. means) or other basic estimates (e.g. regression coefficient) AND variation (e.g. standard deviation) or associated estimates of uncertainty (e.g. confidence intervals)
☐	☒	For null hypothesis testing, the test statistic (e.g. F, t, r) with confidence intervals, effect sizes, degrees of freedom and P value noted<br>*Give P values as exact values whenever suitable.*
☒	☐	For Bayesian analysis, information on the choice of priors and Markov chain Monte Carlo settings
☒	☐	For hierarchical and complex designs, identification of the appropriate level for tests and full reporting of outcomes
☒	☐	Estimates of effect sizes (e.g. Cohen's d, Pearson's r), indicating how they were calculated

Our web collection on statistics for biologists contains articles on many of the points above.

Software and code

Policy information about availability of computer code

Data collection	Microsoft Excel was used to collect data
Data analysis	GraphPad Prism 8.0 (GraphPad Software, San Diego, CA, USA) was used for data analysis, FlowJo software (Tree Star, Ashland, OR, USA) was used to analyze flow data. A free Immune Epitope Database (www.IEDB.org) was used to analyze T cell epitopes with RSV preF protein.

For manuscripts utilizing custom algorithms or software that are central to the research but not yet described in published literature, software must be made available to editors and reviewers. We strongly encourage code deposition in a community repository (e.g. GitHub). See the Nature Portfolio guidelines for submitting code & software for further information.

Data

Policy information about availability of data

All manuscripts must include a data availability statement. This statement should provide the following information, where applicable:

- Accession codes, unique identifiers, or web links for publicly available datasets
- A description of any restrictions on data availability
- For clinical datasets or third party data, please ensure that the statement adheres to our policy

The data that support the findings of this study are available from the corresponding author upon reasonable request.

Human research participants

Policy information about studies involving human research participants and Sex and Gender in Research.

Reporting on sex and gender	*Use the terms sex (biological attribute) and gender (shaped by social and cultural circumstances) carefully in order to avoid confusing both terms. Indicate if findings apply to only one sex or gender; describe whether sex and gender were considered in study design whether sex and/or gender was determined based on self-reporting or assigned and methods used. Provide in the source data disaggregated sex and gender data where this information has been collected, and consent has been obtained for sharing of individual-level data; provide overall numbers in this Reporting Summary. Please state if this information has not been collected. Report sex- and gender-based analyses where performed, justify reasons for lack of sex- and gender-based analysis.*
Population characteristics	*Describe the covariate-relevant population characteristics of the human research participants (e.g. age, genotypic information, past and current diagnosis and treatment categories). If you filled out the behavioural & social sciences study design questions and have nothing to add here, write "See above."*
Recruitment	*Describe how participants were recruited. Outline any potential self-selection bias or other biases that may be present and how these are likely to impact results.*
Ethics oversight	*Identify the organization(s) that approved the study protocol.*

Note that full information on the approval of the study protocol must also be provided in the manuscript.

Field-specific reporting

Please select the one below that is the best fit for your research. If you are not sure, read the appropriate sections before making your selection.

☒ Life sciences ☐ Behavioural & social sciences ☐ Ecological, evolutionary & environmental sciences

For a reference copy of the document with all sections, see nature.com/documents/nr-reporting-summary-flat.pdf

Life sciences study design

All studies must disclose on these points even when the disclosure is negative.

Sample size	All animal experiments were performed with female Balb/c with 5-6 mice/group, all data were analyzed using One-way ANOVA or Two-way ANOVA
Data exclusions	No data was excluded in any experiments.
Replication	All experiments were repeated twice or thrice, all data could be reproduced.
Randomization	All experiment animals including viral challenged mice were divided into groups randomly.
Blinding	This study was an animal experimental study to investigate a mechanism of a T cell epitope located within the preF of RSV, which could boost anti-G of RSV antibody response. blinding was not required and was not performed.

Reporting for specific materials, systems and methods

We require information from authors about some types of materials, experimental systems and methods used in many studies. Here, indicate whether each material, system or method listed is relevant to your study. If you are not sure if a list item applies to your research, read the appropriate section before selecting a response.

Materials & experimental systems

n/a	Involved in the study	
☐	☒	Antibodies
☐	☒	Eukaryotic cell lines
☒	☐	Palaeontology and archaeology
☐	☒	Animals and other organisms
☒	☐	Clinical data
☒	☐	Dual use research of concern

Methods

n/a	Involved in the study	
☒	☐	ChIP-seq
☐	☒	Flow cytometry
☒	☐	MRI-based neuroimaging

Antibodies

Antibodies used	anti-CD4-PECy7 (eBioscience, 25-0041-82), anti-B220-Pacific Blue (Biolegend, 103227), anti-GL-7-PE (Biolegend, 144608), anti-CD138-APC (Biolegend, 142506), anti-IgM-APC-Cy7 (Biolegend, B194508), anti-IgD-FITC (Biolegend, B147607), anti-CD69-PECy7 (Biolegend, 25-0691-81), and anti-CD80-BV605 (eBioscience, 63-0801-82) , anti-Foxp3-APC (eBioscience, 48-5773-80), Anti-IFN-γ-BV421 (Biolegend, 505830) , anti-CD4-PECy7 (eBioscience, 25-0041-82) ,
Validation	Several repeated tests for antibodies were performed with positive and negative controls for insure their qualities.

Eukaryotic cell lines

Policy information about cell lines and Sex and Gender in Research

Cell line source(s)	CHO-K1 cell was used and derived from ATCC
Authentication	N/A
Mycoplasma contamination	Negative when used for the experiments
Commonly misidentified lines (See ICLAC register)	N/A

Animals and other research organisms

Policy information about studies involving animals; ARRIVE guidelines recommended for reporting animal research, and Sex and Gender in Research

Laboratory animals	BALB/c female mice aged at 6-8 week-old were used.
Wild animals	N/A
Reporting on sex	All BALB/C mice involved in the study were females.
Field-collected samples	All BALB/C mice involved in the study were maintained under specific pathogen-free conditions.
Ethics oversight	All animal experiments were approved by the Committee of Experimental Animals of Shanghai Medical College (SHMC), and carried out in compliance with the ARRIVE guidelines.

Note that full information on the approval of the study protocol must also be provided in the manuscript.

Flow Cytometry

Plots

Confirm that:

☒ The axis labels state the marker and fluorochrome used (e.g. CD4-FITC).

☒ The axis scales are clearly visible. Include numbers along axes only for bottom left plot of group (a 'group' is an analysis of identical markers).

☒ All plots are contour plots with outliers or pseudocolor plots.

☒ A numerical value for number of cells or percentage (with statistics) is provided.

Methodology

Sample preparation	ingle cell suspensions were prepared from spleens or lymph nodes of immunized mice after erythrocyte lysis. Cells were counted and stained with Fixable Viability Dye eFluor™ 780 (eBioscience, OR, USA) to remove dead cells before antibody staining. Cells were incubated in PBS (containing 2% FBS) with the following anti-mouse antibodies (all at 1:200 dilution): anti-CD4-PECy7 (eBioscience, 25-0041-82), anti-B220-Pacific Blue (Biolegend, 103227), anti-GL-7-PE (Biolegend, 144608), anti-CD138-APC (Biolegend, 142506), anti-IgM-APC-Cy7 (Biolegend, B194508), anti-IgD-FITC (Biolegend, B147607), anti-CD69-PECy7 (Biolegend, 25-0691-81), and anti-CD80-BV605 (eBioscience, 63-0801-82) for 15 min at room temperature for analyzing the germinal center B cells (B220+ GL7+), plasma (B220- CD138+), Ig switched B cell (B220+ IgM- IgD-) and MFI of CD80 on the activated B cells. For Foxp3 analysis, cells were fixed and permeabilized with a Foxp3/Transcription Factor Staining Buffer Set (eBioscience, 00-5523-00) following the manufacturer's protocol. After fixation, intracellular Foxp3 was labeled by anti-Foxp3-APC (eBioscience, 48-5773-80) in undiluted permeabilization buffer for 1 h at room temperature.
Instrument	LSRFortessa flow cytometry (BD)
Software	FlowJo and Excel

Cell population abundance	The post-sorting cell was labelled with CD4-PECy7, detected the purity by FCM. The results showed that the purified CD4 T cells were about 90%.
Gating strategy	As shown in supplementary Figure 1, Treg cell was the first identified by forward and side scatter profiles, and the forward scatter area versus height was used to select single cells. Selected cells for the absence of staining with a viability dye, followed by gating on the CD3+ cell. Subsequently, CD4+ T cell subpopulation was identified and evaluated for the presence of FOXP3.<br>B cell was identified as above<br>B cells were identified from viable cells as described above, then B220-CD138+ cell was identified. Other B cells were identified by detecting their markers on B220+ cells.

☒ Tick this box to confirm that a figure exemplifying the gating strategy is provided in the Supplementary Information.
